# Comparative genomic analysis of *Acanthamoeba* from different sources and horizontal transfer events of antimicrobial resistance genes

**DOI:** 10.1128/msphere.00548-24

**Published:** 2024-10-01

**Authors:** Xinyi Ling, Xiaobin Gu, Yue Shen, Chunyan Fu, Yumei Zhou, Yiling Yin, Yanqiu Gao, Yiwei Zhu, Yongliang Lou, Meiqin Zheng

**Affiliations:** 1 Wenzhou Key Laboratory of Sanitary Microbiology, Key Laboratory of Laboratory Medicine, Ministry of Education, School of Laboratory Medicine and Life Science, Wenzhou Medical University, Wenzhou, Zhejiang, China; 2 National Clinical Research Center for Ocular Diseases, Eye Hospital, Wenzhou Medical University, Wenzhou, Zhejiang, China; 3 Eye Hospital, School of Ophthalmology and Optometry, Wenzhou Medical University, Wenzhou, Zhejiang, China; University of California, Davis, Davis, California, USA

**Keywords:** *Acanthamoeba*, comparative genomics, antibiotic resistance, horizontal gene transfer

## Abstract

*Acanthamoeba* species are among the most common free-living amoeba and ubiquitous protozoa, mainly distributed in water and soil, and cause *Acanthamoeba* keratitis (AK) and severe visual impairment in patients. Although several studies have reported genomic characteristics of *Acanthamoeba*, limited sample sizes and sources have resulted in an incomplete understanding of the genetic diversity of *Acanthamoeba* from different sources. While endosymbionts exert a significant influence on the phenotypes of *Acanthamoeba*, including pathogenicity, virulence, and drug resistance, the species diversity and functional characterization remain largely unexplored. Herein, our study sequenced and analyzed the whole genomes of 19 *Acanthamoeba* pathogenic strains that cause AK, and by integrating publicly available genomes, we sampled 29 *Acanthamoeba* strains from ocular, environmental, and other sources. Combined pan-genomic and comparative functional analyses revealed genetic differences and evolutionary relationships among the different sources of *Acanthamoeba*, as well as classification into multiple functional groups, with ocular isolates in particular showing significant differences that may account for differences in pathogenicity. Phylogenetic and rhizome gene mosaic analyses of ocular *Acanthamoeba* strains suggested that genomic exchanges between *Acanthamoeba* and endosymbionts, particularly potential antimicrobial resistance genes trafficking including the *adeF*, *amrA*, and *amrB* genes exchange events, potentially contribute to *Acanthamoeba* drug resistance. In conclusion, this study elucidated the adaptation of *Acanthamoeba* to different ecological niches and the influence of gene exchange on the evolution of ocular *Acanthamoeba* genome, guiding the clinical diagnosis and treatment of AK and laying a theoretical groundwork for developing novel therapeutic approaches.

**IMPORTANCE:**

*Acanthamoeba* causes a serious blinding keratopathy, *Acanthamoeba* keratitis, which is currently under-recognized by clinicians. In this study, we analyzed 48 strains of *Acanthamoeba* using a whole-genome approach, revealing differences in pathogenicity and function between strains of different origins. Horizontal transfer events of antimicrobial resistance genes can help provide guidance as potential biomarkers for the treatment of specific *Acanthamoeba* keratitis cases.

## INTRODUCTION

*Acanthamoeba* is an opportunistic protozoan that exists ubiquitously in the natural environment and causes opportunistic infections (1). Pathogenic species can cause blinding *Acanthamoeba* keratitis (AK), as well as fatal granulomatous amebic encephalitis, and skin and lung infections. The life cycle of *Acanthamoeba* typically encompasses two stages: the trophozoite stage in the nutritional form and the resistant cyst stage in the persistent stage (2). Under favorable growth conditions, trophozoites selectively feed on bacteria, fungi, algae, yeasts, or small organic particles through phagocytosis and pinocytosis (3–6). This ability allows them to regulate microbial populations, participating in nutrient conversion and energy metabolism within nature’s ecosystem. Through prolonged interaction and selective evolution, certain microorganisms have evolved resistance to *Acanthamoeba*’s phagocytic activity, sustaining intracellular growth, replication, and establishing symbiotic or parasitic relationships (7–9). Microbiomes that coexist or parasitize within *Acanthamoeba* are known as endosymbionts, which include bacteria, fungi, and giant viruses (10). *Acanthamoeba* has become a replicative niche and dispersal vector for a growing array of pathogenic human pathogens, earning the moniker “Trojan horse” in the microbial world (11). Under adverse environmental conditions, including nutrient deprivation, extreme osmotic pressure, temperature fluctuations, clinical treatment, or drug pressure, the protozoan can transition from a trophozoite form into a double-walled cyst (12). Based on the complete 18S ribosomal RNA (18S rRNA) genotyping analysis, *Acanthamoeba* has been classified into 23 distinct genotypes (T1–T23) (13–17). The T4 genotype is the most prevalent in infections, and studies have suggested that its dominance in human infections stemmed from increased cytotoxicity, reduced therapeutic susceptibility, and greater transmissibility (2, 18). In-depth genomic analysis might elucidate why certain genotypes exhibit greater prevalence in human infections compared to others by exploring their genetic characteristics and resistance profiles.

AK, a rare infectious disease of the cornea attributable to various *Acanthamoeba* genotypes and strains, has seen an increased incidence since Naginton et al. first reported it in 1974 (19). There are currently some limitations regarding AK. Firstly, during the initial stages, AK is easily mistaken for Herpes simplex keratitis, while in its advanced stages, it resembles fungal keratitis or corneal ulcers, leading to potential misdiagnosis (20). Meanwhile, no definitive method or single drug can eradicate both the cystic and trophozoite forms of AK; however, eliminating the trophozoite form proves comparatively more feasible (21–25). Finally, despite previous studies on *Acanthamoeba* genomics, which include proteomic profiling, functional analyses and comparisons across different sources are currently limited, leaving many undiscovered open reading frames (ORFs) within the genome.

Furthermore, since the initial report in 1975 on bacterial survival and reproduction as endosymbionts within *Acanthamoeba* (26), and the discovery of *Acanthamoeba* as a host for pathogenic microorganisms in 1978 (27), extensive studies with sequencing data have highlighted the diversity and abundance of the microbiomes within *Acanthamoeba*. However, limited studies have yet to fully elucidate the specific functional impacts of these endosymbionts on *Acanthamoeba*. Moreover, the molecular and chemical basis of the causal relationship between *Acanthamoeba* phenotypes and endosymbionts’ structure and function remained unknown. Consequently, although horizontal gene transfers (HGTs) between *Acanthamoeba* and its endosymbionts have been documented and were believed to impact both entities’ evolution (28–30), studies elucidating the patterns and functional significance of these HGTs remained scarce. This study aimed to investigate the species diversity of endosymbionts within ocular *Acanthamoeba* and their functional potential on the occurrence and development of AK, providing valuable insights for clinical diagnosis and treatment strategies for AK.

In our study, whole-genome sequencing (WGS) analysis provided detailed insights into 19 AK pathogenic strains, encompassing genomic characteristics and phylogenetic relationships. With the integration of 29 publicly available genomes, we obtained sequences for 48 *Acanthamoeba* strains from eyes, the environment, and other sources. We then conducted a comprehensive functional analysis of *Acanthamoeba*’s “open” state from these different sources, exploring the influence of various host ecological niches on *Acanthamoeba*’s genetic diversity. Our study also furnished a wealth of evidence suggesting that horizontal transfer of antimicrobial resistance genes (ARGs) originating from *Burkholderia* may augment *Acanthamoeba*’s resistance to neomycin and azithromycin, macrolide antibiotics.

## RESULTS

### Phylogenetic analysis and general genomic characteristics of 19 AK isolates

**Fig 1:**
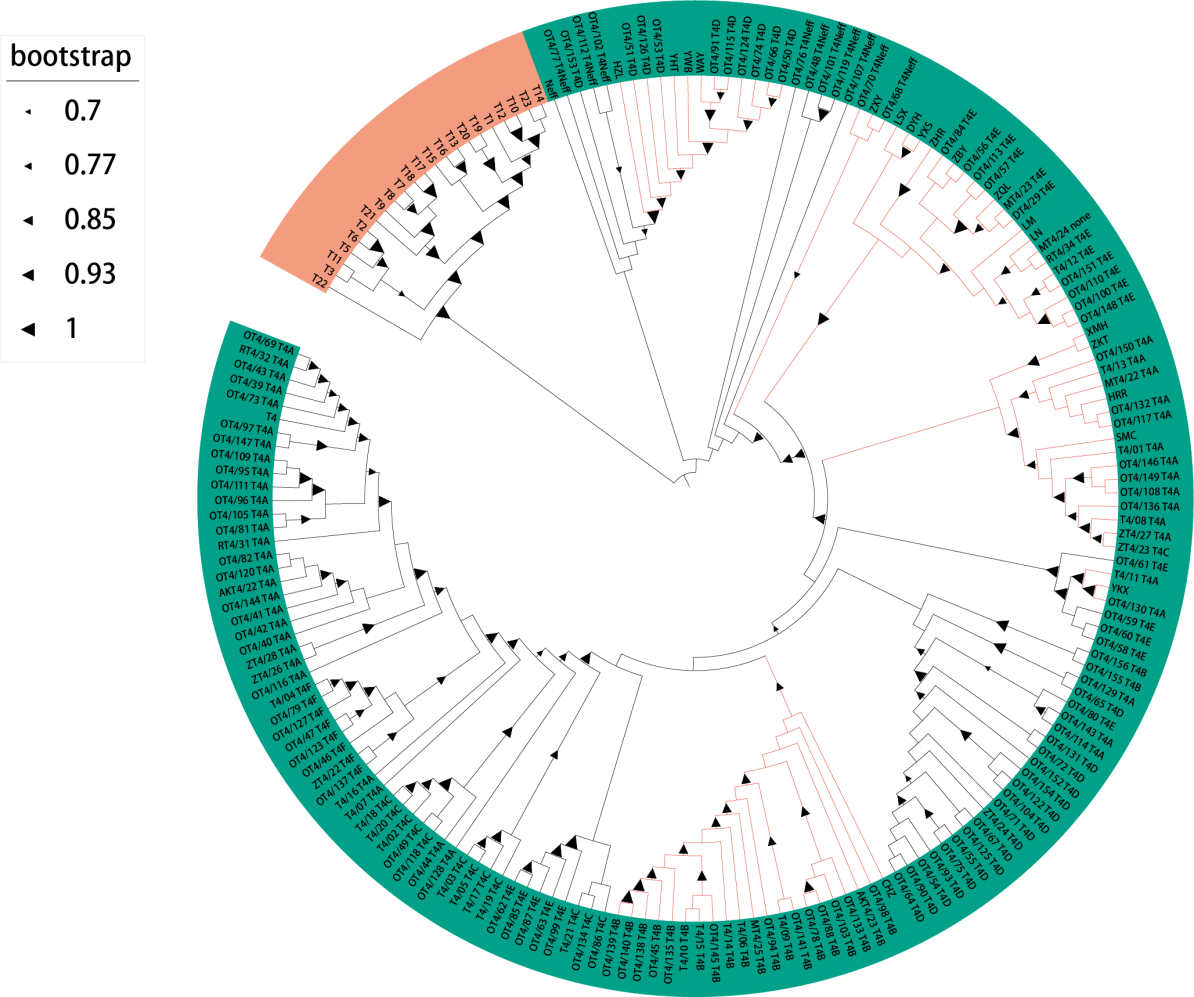
Phylogenetic maximum likelihood tree based on 18S rRNA. The green branch represents T4 genotype, while T1–T3, T5–T23 genotypes are in orange. The 19 ocular isolates we identified are marked with colored lines. Main genotypes are included: T1: *A. castellanii* V006 (U07400); T2: *A. palestinensis Reich* (U07411); T3: *A. griffini* H37 (S81337); T4: *A. castellanii* (U07413); T5: *A. lenticulata* E18-2 (U94735); T6: *A. palestinensis* 2802 (AF019063); T7: *A. astronyxis* R&H (AF019064); T8: *A. tubiashi* OC-15C (AF019065); T9: *A. comandoni* (AF019066); T10: *A. culbertsoni* Lilly A1 (AF019067); T11: *A. hatchetti* BH-2 (AF019068); T12: *A. healyi* (AF019070); T13: *Acanthamoeba* sp. UWC9 (AF132134); T14: *Acanthamoeba* sp. PN15 (AF333607); T15: *A. jacobsi* AC305 (AY262365); T16: *Acanthamoeba* sp. (GQ380408.2); T17: *Acanthamoeba* sp. (GU808301.1); T18: *A. byersi* (MN153028.1); T19: *Acanthamoeba* sp. (DQ451163.2); T20: *Acanthamoeba* sp. (KR780564.1); T21: *A. pyriformis* (KX840327.1); T22: *A. royreba* ATCC30884 (CDEZ01000000); T23: *Acanthamoeba* sp. (MZ272148.1)

The analysis of the 18S rRNA sequences showed a high degree of homology, ranging from 98.77% to 100%, with *Acanthamoeba* species. To elucidate the phylogenetic relationships, a phylogenetic tree was constructed based on a comparative analysis of the 18S rRNA sequences. This finding revealed that all 19 pathogenic strains belonged to the T4 genotype, further subclassified into five subtypes: T4A (HRR, SMC, XMH, YKX, ZKT), T4B (CHZ), T4D (HZL, WAY, YHT, YWB), T4E (DYH, LM, LN, LSX, YXS, ZQL, ZHR, ZBY), and T4Neff (ZXY) (Fig. 1). This was consistent with the study that the T4 genotype was the main genotype associated with AK, highlighting the diversity and potential pathogenicity within this group (31).

**Fig 2:**
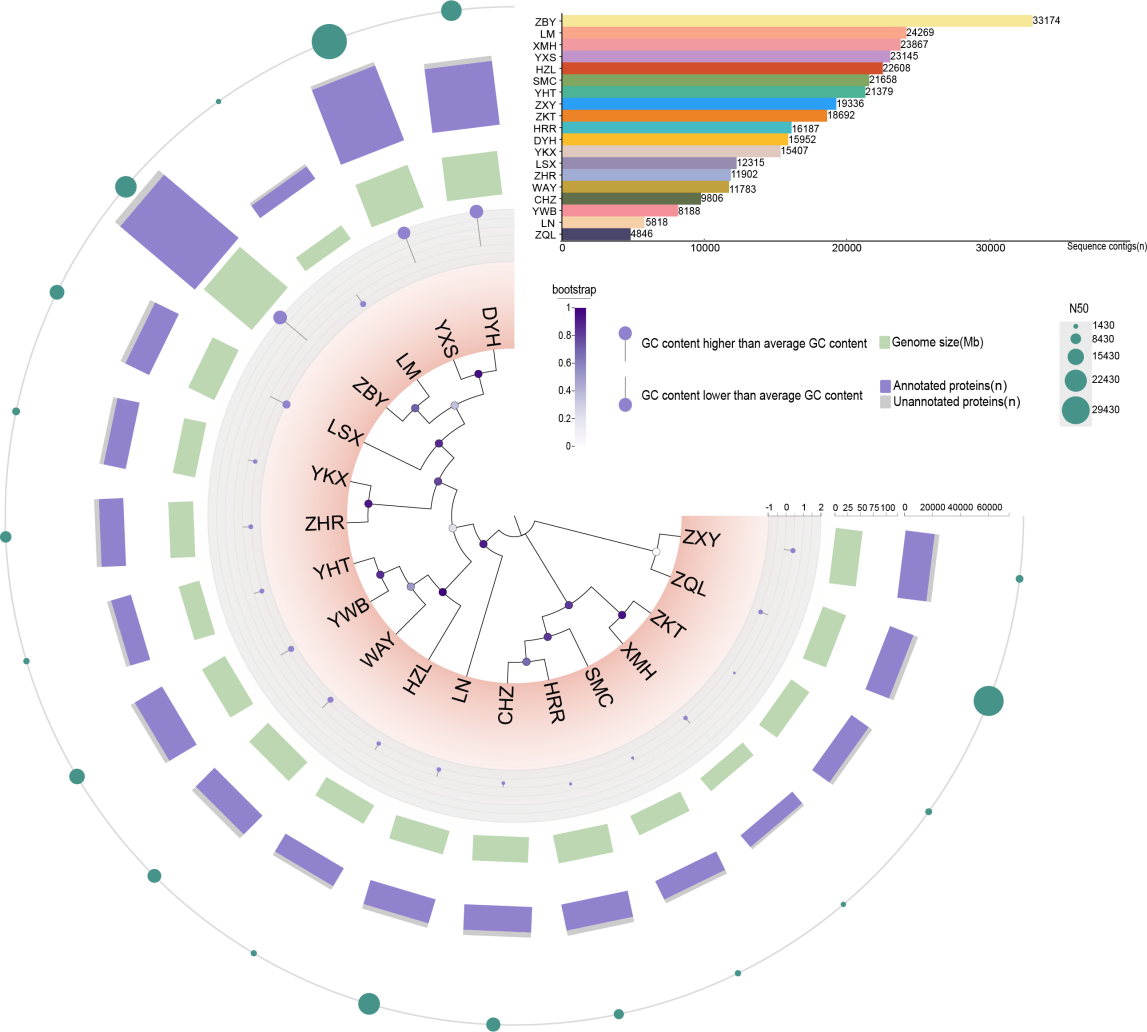
Genome characteristics of 19 ocular *Acanthamoeba* isolates. Circle from the outer to the inner: (1) N50 (a metric for assessing the quality of genome assembly), (2) annotated proteins and unannotated proteins number, (3) genome size (Mbp), (4) GC content (in comparison to the average G + C content, outward/inward facing circle indicates higher or lower content of G + C), (5) phylogenetic tree based on the core genomes. In the upper right corner of the circle are bar graphs representing the sequence contigs of the 19 ocular strains and legends, respectively.

High-throughput sequencing was conducted on the 19 pathogenic AK strains to analyze their genomic characteristics. The genomic characteristics and detailed information were provided in Table S1. Estimated draft genome sizes for the *Acanthamoeba* isolates ranged from 32.01 to 122.47 Mbp, with predicted protein counts between 12,758 and 74,829, and an average gene content of 58.02% (Fig. 2). The LM and ZBY strains represented the minimum and maximum ranges of genome size and predicted proteins, respectively. In part, a bigger genome means more quantity of proteins. Comparison of genome sizes with other *Acanthamoeba* in public databases revealed similarities, suggesting the representativeness of the 19 sequenced data sets. Additionally, gene content, predicted protein counts, and protein annotations for the isolates aligned with those in public databases, revealing a common genetic relationship.

### Pan-genomic analysis and functional characterization of *Acanthamoeba* from different sources

**Fig 3:**
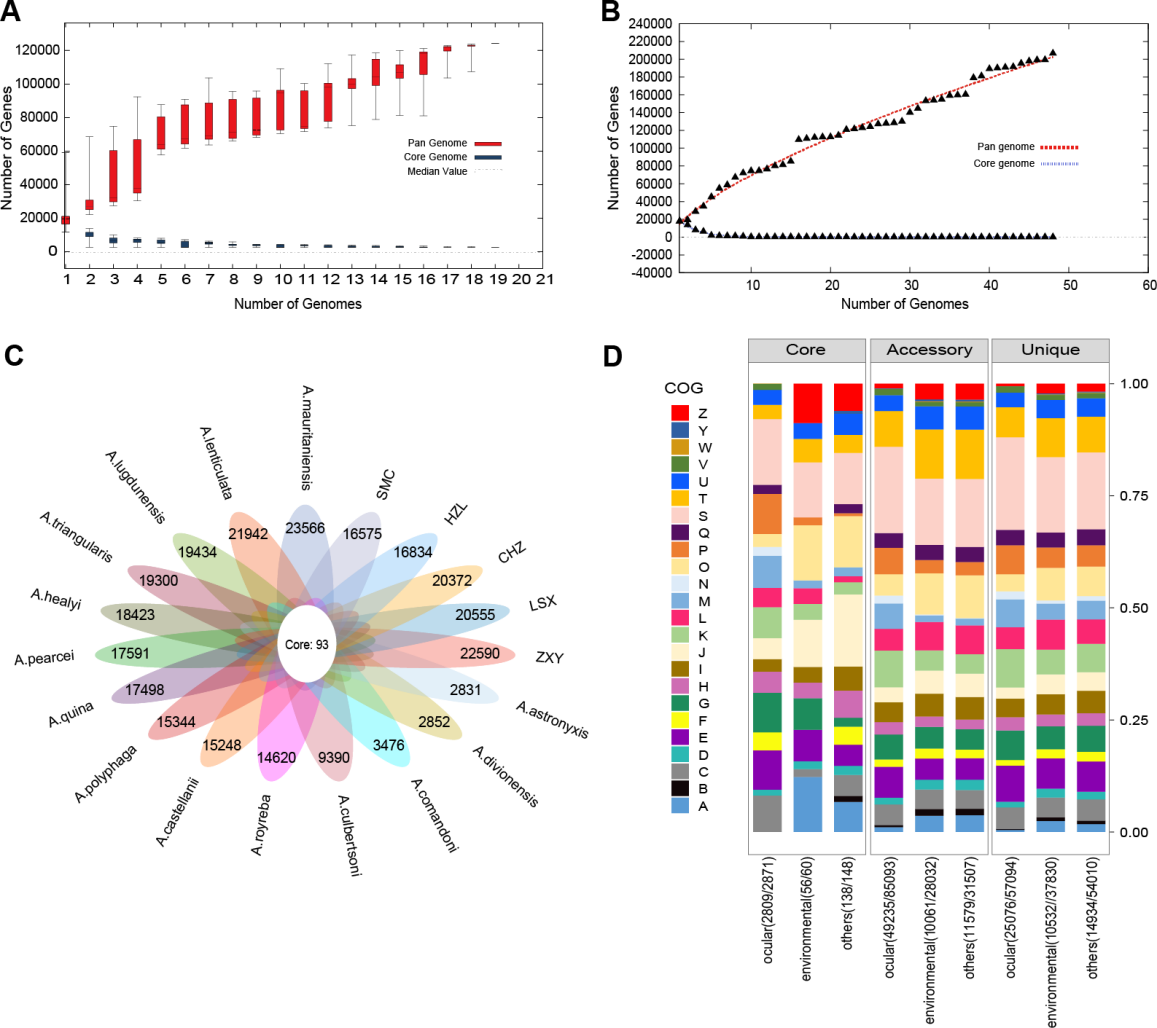
Pan-genome analysis, flower plot of distinct *Acanthamoeba* species, and clusters of orthologous group (COG) functional annotations of *Acanthamoeba* from different sources. (**A**) Gene accumulation curves showing general trends for pan (red) and core (blue) genomes of 19 ocular *Acanthamoeba* strains. (**B**) Gene accumulation curves displaying general trends for pan (red) and core (blue) genomes of collected strains. (**C**) Flower plot of distinct *Acanthamoeba* species core and pan genomes. (**D**) COG function annotations from the pan-genomes within strains from different sources. Involved COG categories are as follows: A, RNA processing and modification; B, chromatin structure and dynamics; C, energy production and conversion; D, cell cycle control, cell division, chromosome partitioning; E, amino acid transport and metabolism; F, nucleotide transport and metabolism; G, carbohydrate transport and metabolism; H, coenzyme transport and metabolism; I, lipid transport and metabolism; J, translation, ribosomal structure, and biogenesis; K, transcription; L, replication, recombination, and repair; M, cell wall/membrane/envelope biogenesis; N, cell motility; O, post-translational modification, protein turnover, chaperones; P, inorganic ion transport and metabolism; Q, secondary metabolites biosynthesis, transport, and catabolism; T, signal transduction mechanisms; U, intracellular trafficking, secretion, and vesicular transport; V, defense mechanisms; W, extracellular structures; S, function unknown; Y, nuclear structure; Z, cytoskeleton.

To identify the pan-genomic characteristics, genetic similarities and variations among 48 strains were determined (32). Gene accumulation curves (Fig. 3A and B) demonstrated that there was an exponential decline in the quantity of core genomes, indicating the representativeness of the strains collected from diverse sources. Meanwhile, the pan-genome data (Fig. 3A and B) showed a power law curve, suggesting a “open” pan-genome in which several new genes were added with each genome sequence, and a rising overall count of *Acanthamoeba* genes as the number of genomes increased.

Detailed analyses examined the distribution and clusters of orthologous group (COG) function of the core genome and pan-genome across 19 distinct *Acanthamoeba* species or subtypes, aiming to elucidate genetic diversity among the species. *Acanthamoeba* pan-genome size reached 298,441 genes encompassing clusters or unique genes, while a total of 93 clusters composed the core genome, which represented 0.03% of the pan-genome (Fig. 3C). The genes with core, association, and uniqueness exhibited substantial disparities. Specifically, Fisher’s exact test (FDR < 0.05) revealed significant enrichment of core genes in translation, ribosomal structure and biogenesis, and in post-translational modification, protein turnover, and chaperones (FDR = 0.005). COG analysis showed that the most represented categories among accessory and unique genes included information storage and processing, cellular processes and signaling, and metabolism functions. Additionally, significant enrichment was observed for accessory genes in coenzyme transport and metabolism (FDR = 0.036), and for unique genes in intracellular trafficking, secretion, and vesicular transport (FDR = 0.008).

Also, all gene clusters were further grouped into COGs to investigate the function of each gene in the pan-genome of each strain from different sources (Fig. 3D). In eye-sourced strains, Fisher’s exact test (FDR < 0.05) revealed that core genes were significantly enriched in 10 of 20 COG categories. These included amino acid transport and metabolism, cell wall/membrane/envelope biogenesis, inorganic ion transport and metabolism, carbohydrate transport and metabolism, nucleotide transport and metabolism, replication, recombination and repair, coenzyme transport and metabolism, lipid transport and metabolism, post-translational modification, protein turnover, chaperones, and translation, ribosomal structure and biogenesis. However, core genes from strains sourced from the environment and other sources were only enriched in translation, ribosomal structure, and biogenesis (FDR < 0.05). Secondly, a comparison of the functional enrichment of accessory genes COG in strains from diverse sources revealed that metabolism and information storage and processing were significantly abundant in the accessory genes of all strains while those from the eye exhibited significant enrichment in cellular processes and signaling. Meanwhile, unique genes from all strains—except those sourced from eyes—were enriched in genes associated with information storage and processing. Moreover, a large number of genes fell into the “unknown function” category across all strains from various sources (Fig. 3D). To summarize, pan-genomic functions differed across strains from various sources, and the abundance of certain gene functions might trigger transmission between strains, potentially influencing their pathogenicity and drug resistance.

**Fig 4:**
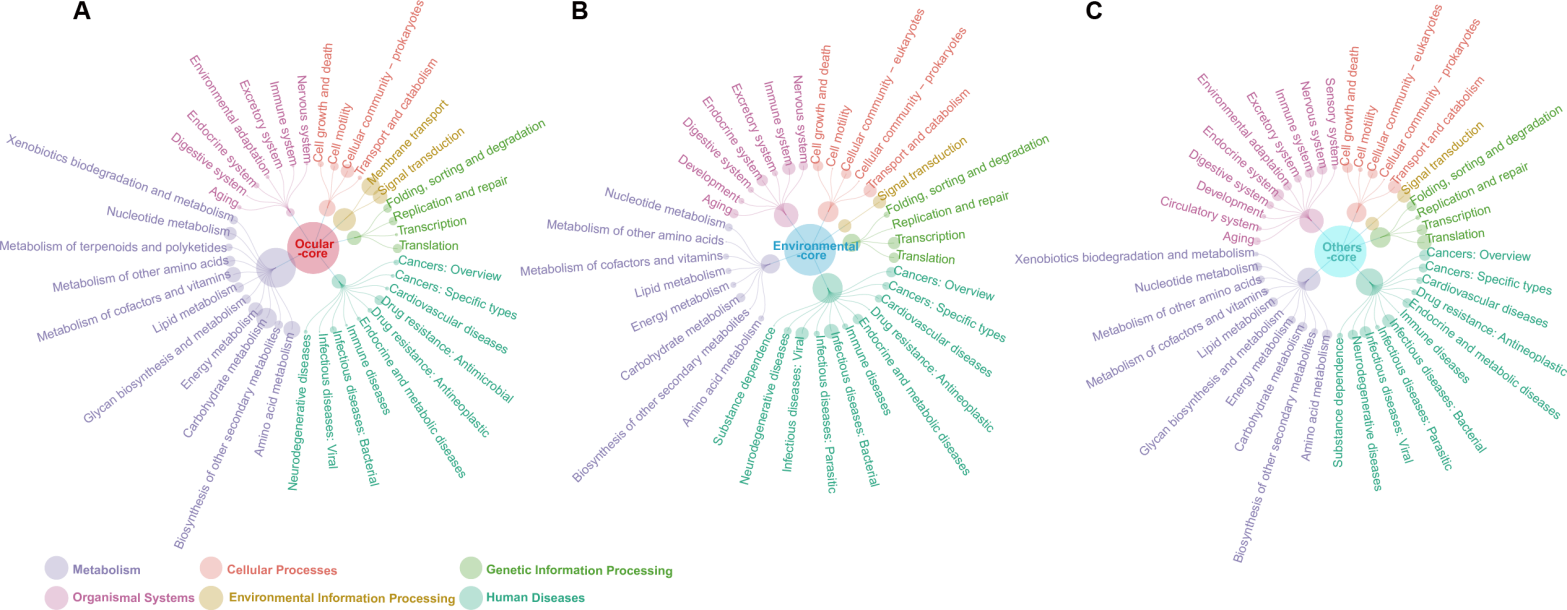
Network diagrams of KEGG functional classification associated with core genes in different sources. From the inner to the outer circle: the number of core genes enriched to each major class of KEGG (six classes in total); the number of core genes enriched to each KEGG pathway.

**Fig 5:**
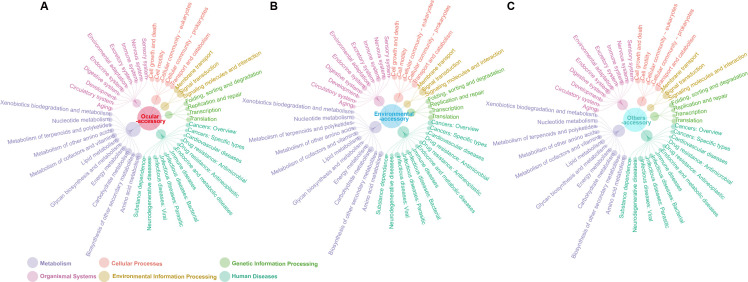
Network diagrams of KEGG functional classification associated with accessory genes in different sources. From the inner to the outer circle: the number of accessory genes enriched to each major class of KEGG (six classes in total); the number of accessory genes enriched to each KEGG pathway.

**Fig 6:**
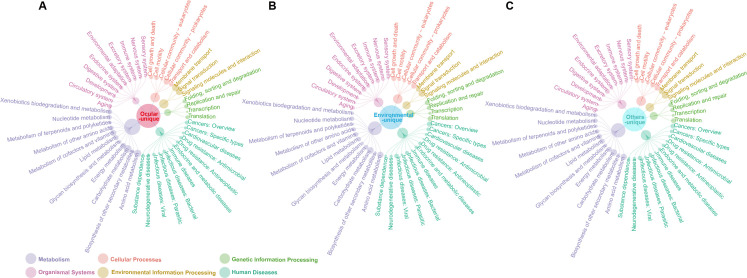
Network diagrams of KEGG functional classification associated with unique genes in different sources. From the inner to the outer circle: the number of unique genes enriched to each major class of KEGG (six classes in total); the number of unique genes enriched to each KEGG pathway.

Furthermore, predicted ORFs within the pan-genome of all strains from various sources were further classified into functional Kyoto Encyclopedia of Genes and Genomes (KEGG) pathways. The numbers represented by each colored sphere are in Table S4. The results revealed that, in total, 1,340/2,871, 41/60, and 91/148 core genes were annotated to KEGG pathways among strains from various sources, including ocular, environmental, and others (Fig. 4A through C). Of the ocular isolates’ core genes, 57.79% were identified as related to metabolism, predominantly carbohydrate metabolism, through KEGG annotation. This was followed by 18.04% linked to environmental information processing and 8.97% to cellular processes. However, the predominant KEGG pathways for core genes from environmental and other sources were human diseases (32.82%, 26.5%), then organismal systems (21.37%, 20.85%), cellular processes (15.27%, 13.43%), and metabolism (12.98%, 18.73%). In the subcategory comparison of KEGG functions, ocular core genes showed no enrichment for cellular community-eukaryotes in cellular processes, infectious diseases: parasitic, substance dependence in human diseases, or development in organismal systems. Additionally, core genes from environmental and other sources lacked functions in membrane transport, environmental information processing, drug resistance (antimicrobial in human diseases), and metabolism of terpenoids and polyketides. For accessory genes annotated to KEGG pathways, the numbers were comparable between the environmental (4,660/28,032) and other groups (5,346/31,507), while the ocular group had 15,882/85,093 (Fig. 5A through C). Regarding the functional distribution of KEGG across different sources, metabolism genes comprised the majority of accessory genes in ocular, environmental, and other sources. Primarily, these genes were involved in carbohydrate and amino acid metabolism, followed by human diseases. The finding revealed that 7,078/57,094, 4,381/37,830, and 5,964/54,010 unique genes were annotated to KEGG pathways in ocular, environmental, and other groups, respectively (Fig. 6A through C). A similar distribution of functions according to KEGG was observed across sources: metabolism genes formed the largest fraction of unique genes specific to ocular, environmental, and other sources, with prominent involvement in carbohydrate and amino acid metabolism. This was followed by cellular processes, and human diseases, respectively. Noteworthy was that, unlike strains from environmental and other sources, the core genes of ocular strains revealed antimicrobial drug-resistant pathways. This phenomenon could stem from exposure to selection pressure from broad-spectrum antibiotics during environmental adaptation and the presence of endosymbionts.

### Antimicrobial resistance across AK pathogenic strains

**Fig 7:**
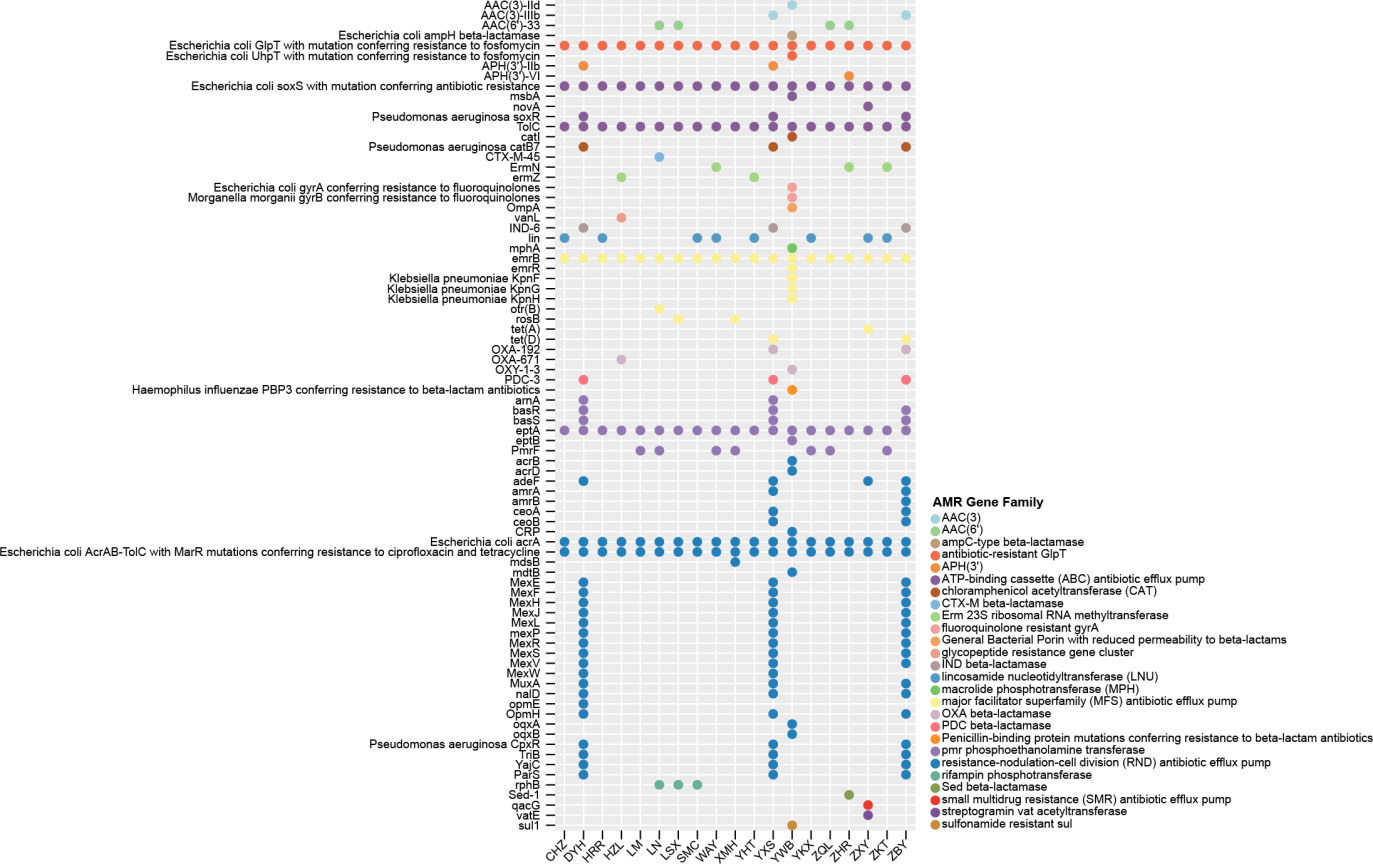
Antimicrobial resistance genes predicted with the CARD database.

The capability of *Acanthamoeba* trophozoites to exhibit antimicrobial resistance and their transformation into drug-resistant cysts during therapy have constricted treatment alternatives, resulting in recurrent clinical infections and complicating the treatment of AK (33, 34). To detect the antibiotic resistance properties of *Acanthamoeba* at the genomic level, ARGs were identified using BLASTp for the Comprehensive Antibiotic Resistance Database (CARD) (35). Our data revealed that seven genes related to antimicrobial resistance (AMR) were shared by all ocular *Acanthamoeba* strains, including *Escherichia coli GlpT* with mutation conferring resistance to fosfomycin, *Escherichia coli soxS* with mutation conferring antibiotic resistance, *TolC*, *emrB*, *eptA*, *Escherichia coli acrA*, and *Escherichia coli AcrAB-TolC* with *MarR* mutations conferring resistance to ciprofloxacin and tetracycline (Fig. 7). *Escherichia coli GlpT* with mutation conferring resistance to fosfomycin develops resistance to phosphonic acid antibiotic through an antibiotic target alteration mechanism, while *Escherichia coli soxS* with mutation conferring antibiotic resistance develops resistance to tetracycline antibiotic through antibiotic efflux, reduced permeability to antibiotic, and antibiotic target alteration mechanisms (36, 37). *TolC* is associated with peptide antibiotics and aminoglycoside antibiotics, which belong to the AMR gene family with ATP-binding cassette antibiotic efflux pump (38). *emrB* confers resistance to fluoroquinolone antibiotics primarily via an antibiotic efflux mechanism (39), while *eptA* is associated with resistance to peptide antibiotics (40). Both *Escherichia coli acrA* and *Escherichia coli AcrAB-TolC* with *MarR* mutations conferring resistance to ciprofloxacin and tetracycline belong to the AMR gene family of resistance-nodulation-cell division (RND) antibiotic efflux pump, which are mainly resistant to tetracycline antibiotics (41, 42). It should be noted that *AAC (3)-IId*, *AAC (3)-IIIb*, *AAC(6′)-33*, *APH(3′)-IIb*, *APH(3')-VI*, *acrD*, *ceoA*, and *ceoB* are characterized by resistance to neomycin; *ErmN*, *ermZ*, *mphA*, *CRP*, *MexJ*, *MexL*, *mexP*, *MexR*, *MexV*, *MexW*, *MuxA*, *nalD*, and *opmE* are characterized by resistance to macrolide. *TolC*, *Klebsiella pneumoniae KpnF*, *Klebsiella pneumoniae KpnG*, *Klebsiella pneumoniae KpnH*, *amrA*, *amrB*, *Pseudomonas aeruginosa CpxR*, and *ParS* are characterized by resistance to neomycin and macrolide, which are used in the treatment of AK (43, 44). And *TolC*, *OmpA*, *Klebsiella pneumoniae KpnF*, *Klebsiella pneumoniae KpnG*, *Klebsiella pneumoniae KpnH*, *rosB*, *arnA*, *basR*, *basS*, *eptA*, *eptB*, *PmrF*, *MexR*, *nalD*, and *Pseudomonas aeruginosa CpxR* are resistant to peptide antibiotics such as polymyxin-B, a commonly used antibiotic in ophthalmology (45). These findings aligned with the information reported in the CARD.

### Taxonomic diversity and horizontal transfer of antimicrobial resistance genes between *Acanthamoeba* and endosymbionts

**Fig 8:**
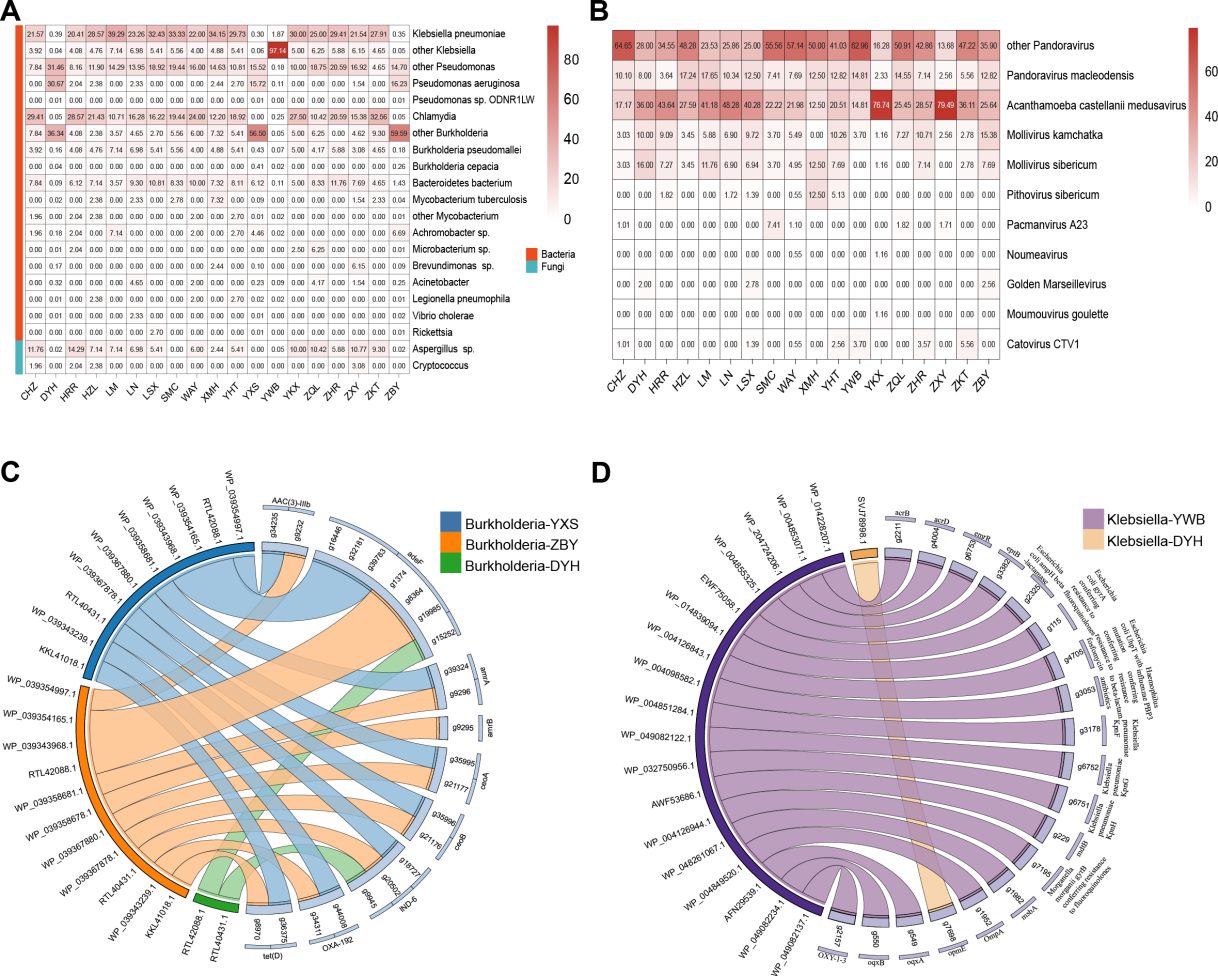
Taxonomic distribution of the predicted endosymbionts proteins in ocular strains and network of isolates with representative homologous ARGs. (**A**) Taxonomic distribution of the predicted bacteria and fungi proteins in ocular strains. The number of predicted proteins was indicated by BLASTp. (**B**) Taxonomic distribution of the predicted viral proteins in ocular strains. The number of predicted proteins was indicated by BLASTp. (**C**) Homologous ARGs of *Burkholderia* and *Acanthamoeba*. The number of exchanged genes for which a homolog was identified in each isolate, represented by different colors. (**D**) Homologous ARGs of *Klebsiella* and *Acanthamoeba*. The number of exchanged genes for which a homolog was identified in each isolate, represented by different colors.

By analyzing the taxonomic distribution results of the best BLASTp searches for the *Acanthamoeba* proteins, we predicted the protein sequences of endosymbionts that best matched the *Acanthamoeba*. Among bacteria and fungi, the endosymbiont genes of *Klebsiella*, *Pseudomonas*, *Burkholderia*, and *Bacteroidetes* were identified in all 19 ocular strains we studied (Fig. 8A). The presence of certain endosymbiont genes, such as those from *Chlamydia* (18/19), *Mycobacterium* (14/19), and *Aspergillus* sp. (17/19), was observed in the majority of the genomes. *Aspergillus* not only causes keratitis but also induces host cell lysis through the phagocytosis of its conidia by *Acanthamoeba*, leading to intracellular germination (46). Conversely, there were instances where certain endosymbiont genes were observed only in a limited number of genomes. It was found that *Acanthamoeba* genes exhibiting the highest correlation with *Legionella pneumophila* sequences occurred in 36.84% (7/19) of the ocular strains. The species was known to induce Legionnaires’ disease and was the first amoeba-resistant bacteria discovered to proliferate within *Acanthamoeba* (47). Furthermore, the shared proteins correlating with the highest scoring matches were found to belong to *Vibrio cholerae* in approximately 21.05% (4/19) of the strains examined; this species parasitizes amoebae within the environment (48) or can survive the environment with amoebae (49), as well as causes varying degrees of gastroenteritis. Regarding giant viruses, genes from endosymbionts including *Pandoravirus* and *Acanthamoeba castellanii medusavirus* were found in 18 ocular isolates (Fig. 8B). They have been shown to have complex patterns of gene transfer, evidence of multidirectional sequence exchange mechanisms, and potential species-specific interactions with *Acanthamoeba* (30).

Interestingly, putative trafficking of ARGs between *Acanthamoeba* and endosymbionts was identified in Table S2 (Fig. 8C and D) including *Burkholderia*, *Klebsiella*, and *Pseudomonas*. A comprehensive characterization of ARGs transfer in *Acanthamoeba* was then conducted, involving *Acanthamoeba* ARGs and the diversity of endosymbionts’ homologous sequences, through multi-alignment and the construction of a phylogenetic tree demonstrating bootstrap values and topologies, to assess potential horizontal transfer events of ARGs between *Burkholderia* and *Acanthamoeba*.

**Fig 9:**
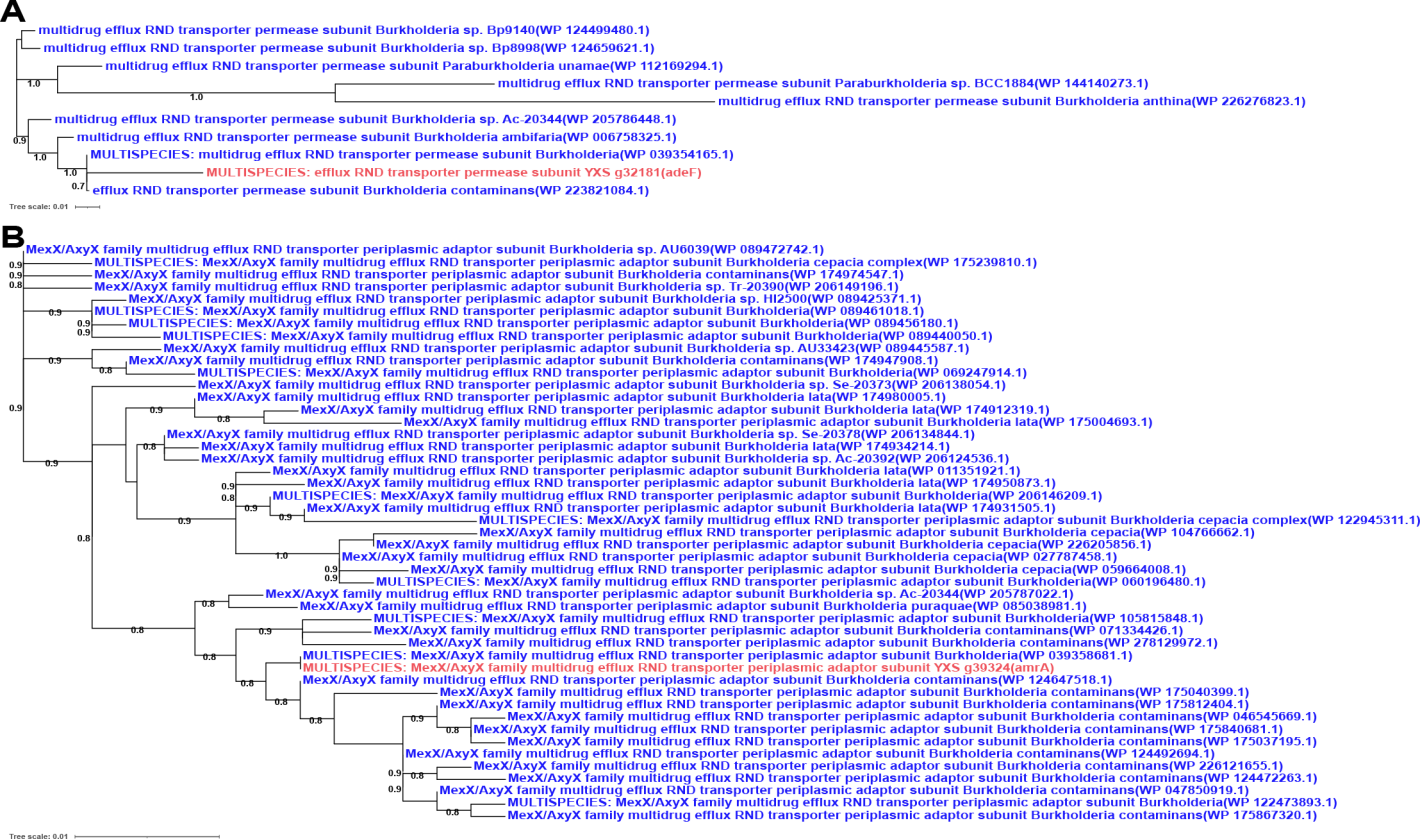
Phylogenetic analysis (**A and B**) of *Acanthamoeba* ARGs. The trees were constructed based on homologous sequences acquired from searching against the nr database by BLASTp and with FastTree. In red: ARG protein of *Acanthamoeba*; in blue: the closest homolog from *Burkholderia*.

**Fig 10:**
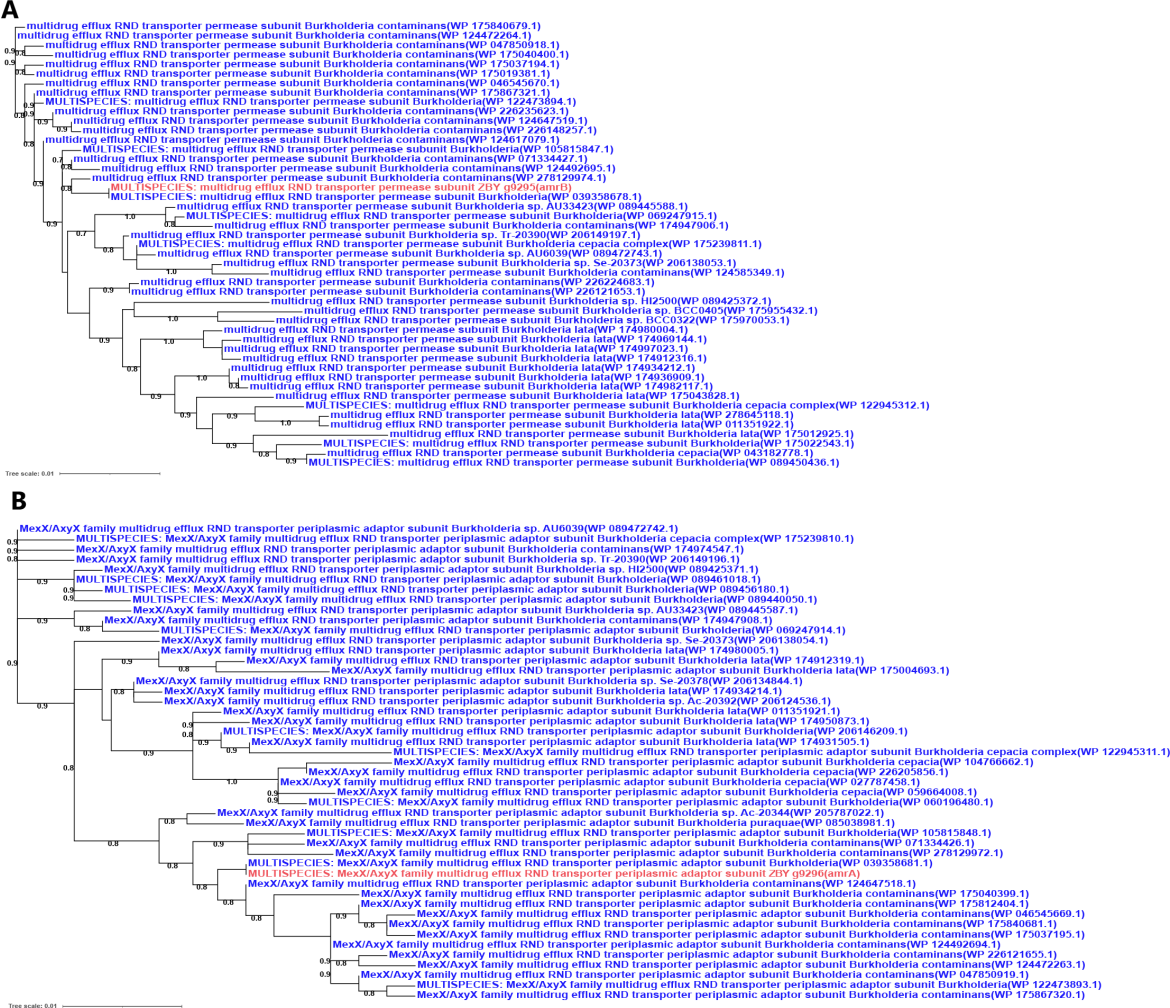
Phylogenetic analysis (**A and B**) of *Acanthamoeba* ARGs. The trees were constructed based on homologous sequences acquired from searching against the nr database by BLASTp and with FastTree. In red: ARG protein of *Acanthamoeba*; in blue: the closest homolog from *Burkholderia*.

**Fig 11:**
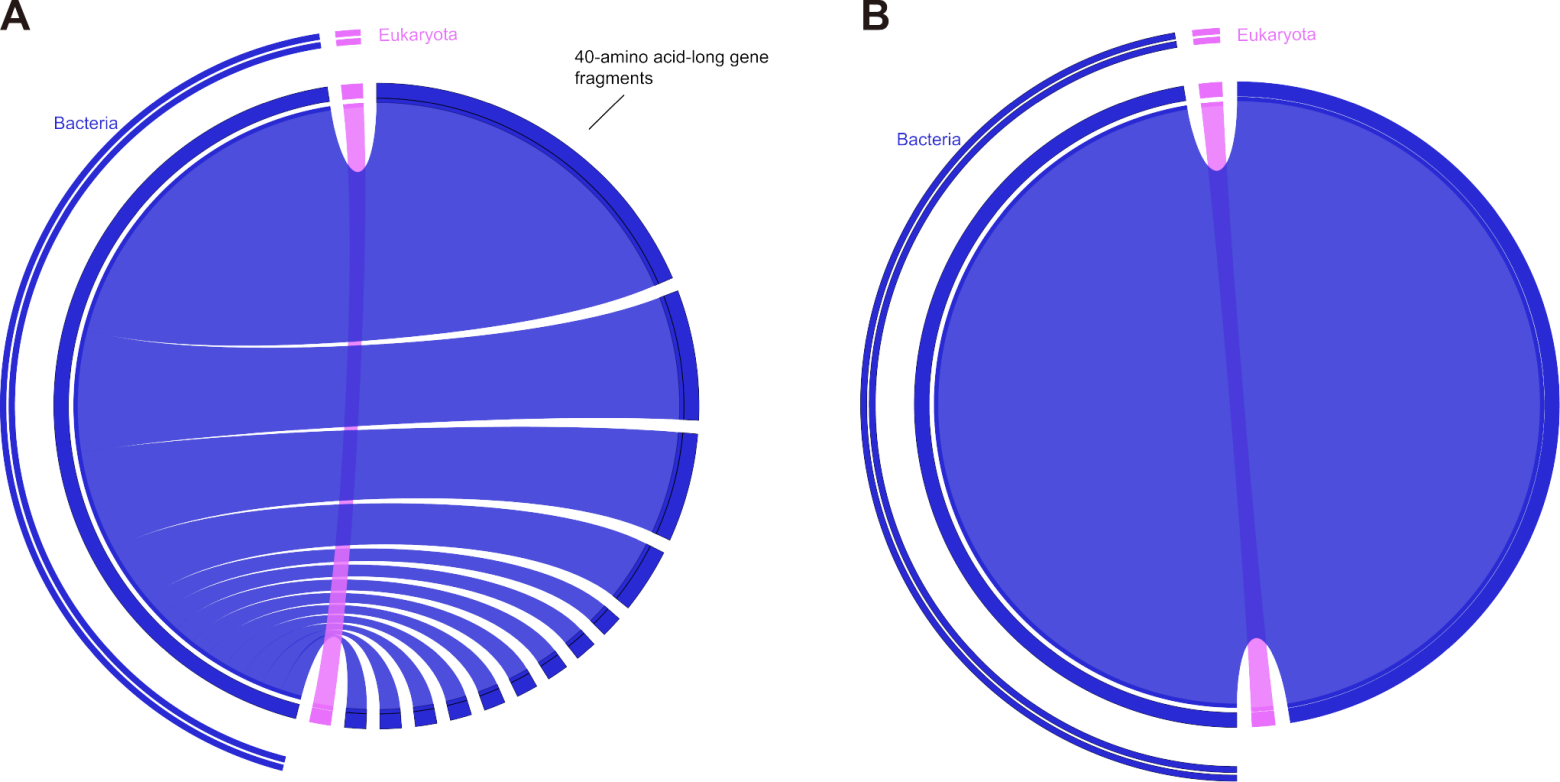
Rhizomes gene mosaicism of *Acanthamoeba* sequences shared homologs with *Burkholderia*. Cases “A” and “B” correspond to the cases shown in Fig. 9A and B. We searched for the 40 best homologous sequences for each *Acanthamoeba* gene sequence and integrated them in a circular visualization.

To minimize false positives, HGTs characterized by unclear and weakly supported phylogenies were manually excluded. For all observed horizontal transfer patterns of ARGs, it was confirmed that 17.39% (4 of 23) of the candidate resistance genes shared with *Burkholderia* were associated with strong bootstrap-supported HGT (Fig. 9 and 10). Specifically, a case of HGT involving the *adeF* gene from *Burkholderia* to *Acanthamoeba* was observed (Fig. 9A); *adeF* contributes to tetracycline and fluoroquinolone antibiotic resistance and is a member of the AMR gene family RND antibiotic efflux pump (50). Additionally, a recent acquisition was recorded involving one *amrB* gene and also two cases of *amrA* gene originating from *Burkholderia* to *Acanthamoeba* (Fig. 9B and 10). Both *amrA* and *amrB* are known to confer resistance to aminoglycoside and macrolide antibiotics and belong to the AMR gene family of RND antibiotic efflux pumps (51). Neomycin and azithromycin, commonly used aminoglycoside and macrolide antibiotics for treating AK, respectively, may be less effective against *Acanthamoeba* strains harboring the horizontally transferred *amrA* and *amrB* genes, thus limiting treatment options (43). Furthermore, the closest homologs of the four *Acanthamoeba* ARGs identified to date originated from *Burkholderia*, and all evidenced unidirectional transfer, namely from *Burkholderia* to *Acanthamoeba*. While the phylogenetic analysis often proved inconclusive regarding the significance of sequence flow, a thorough examination of the predicted sequence in the ARGs for each of the four previously described situations was conducted. This included a mosaic approach to more comprehensively demonstrate the most similar homologs associated with the *Acanthamoeba* ARGs (Fig. 11). Finally, it was observed that in all instances of sequence mosaicing, the most closely matched homologous sequences originated from *Burkholderia* (Fig. 11).

## DISCUSSION

*Acanthamoeba* serves as vector and reservoir for pathogens and is the most prevalent pathogen causing AK (10, 52). Currently, there is a lack of a thorough understanding of the genomes of *Acanthamoeba* strains from various sources. Consequently, this study aimed to uncover the genetic variations among *Acanthamoeba* strains from diverse sources, including ocular, environmental, and others, employing WGS techniques. To our knowledge, this is the largest collection of *Acanthamoeba* genome data sets from different sources, with data sources including patients with corneal AK ulcers and the public database.

In our study, we gained insight into the phylogenetic relationships and genomic features of ocular-sourced *Acanthamoeba* by gathering, sequencing, and analyzing 19 genomes. The isolates were identified as *Acanthamoeba* species using BLASTn software, followed by the construction of a phylogenetic tree based on 18S rRNA gene sequence analysis. During the biological evolution of *Acanthamoeba*, mutations in certain genes could have triggered pathogenicity, with specific 18S rRNA genotypes displaying a propensity for corneal keratophilicity and a higher likelihood of causing corneal infections. Conversely, another study found no significant correlation between specific genotypes or species and the severity of AK or its response to drug therapy (53). Therefore, further research involving a larger sample size of clinical specimens is warranted to establish a more definitive correlation between genotyping and clinical outcomes. In terms of distribution, Corsaro and Venditti (54) recently identified T4H as a novel subtype of the T4 category (54). Future research will focus on identifying virulence profiles and genetic markers specific to certain genotypes, potentially clarifying distribution puzzles. However, to some extent, ribosomal typing may be limiting, with whole-genome analysis providing more comprehensive information. Our study sequenced 19 ocular-sourced *Acanthamoeba* genomes and obtained high-quality baseline data on genome size, gene content, and the number of predicted and annotated proteins, which facilitated subsequent complete and precise genome analysis.

Pan-genomic analyses provided unique biological insights into the taxonomic pedigree (55), genetic variation (56), pathogenesis (57), HGT (58), and niche adaptation of pathogens (57). Pan-genomic analysis of 48 genomes from different sources determined that *Acanthamoeba* had an open pan-genomic structure, implying that these *Acanthamoeba* strains can colonize and utilize a variety of ecological niches. *Acanthamoeba* and endosymbionts were examples of organisms whose symbiotic lifestyle meant that there was HGT activity between them, enabling access to new genetic material and the ability to survive in complex host environments (59). Furthermore, the core genome’s minimal proportion (0.03%) about the pan-genome underscored the *Acanthamoeba* genome’s adaptability. To better understand functional distributions, this research provided further insight into the characteristic survival patterns of *Acanthamoeba* from various isolated sources, which have been categorized into multiple groups, particularly ocular isolates that exhibited significant differences. Of course, more data on experiments and pathogenicity are needed for in-depth analysis in the future, combined with clinical case outcomes.

Homology-based proteome searches highlighted the potential for varied contributions to the genome. Bacteria that were endosymbionts in *Acanthamoeba* have been reported in both clinical and environmental specimens (60–62). Our current understanding of the parasitic mechanisms between endosymbionts and amoebae remained limited. The proliferation of *L. pneumophila* in amoebae depended on the Icm/Dot type IV secretion system, which played a pivotal role in various biological processes including enhancement of phagocytosis, modulation of membrane pore formation and their release from host cells, acquisition of rough endoplasmic reticulum for the phagosome, inhibition of phagosome–lysosome fusion, as well as induction of caspase-3 dependent apoptotic host cell death, and release (63). These findings suggested that other intracellular endosymbionts may employ similar molecular mechanisms to ensure survival within amoebae.

A growing body of data suggested that vertical inheritance and horizontal gene exchange jointly triggered genome evolution in space and time. Distinguished from the vertical inheritance, which involved the transmission of genes from parents to offspring, HGT was a common and significant genetic pathway, conferring new biological functions on the host, particularly for microorganisms within large communities sharing a given ecological niche (64, 65). As predicted by the “melting pot” hypothesis, amoeba could act both as fertile grounds for genetic exchange between endosymbionts and as participants in HGTs, such as with intracellular bacteria and giant viruses (28, 66, 67). Studies have shown that ubiquitous and recurrent HGT between *Acanthamoeba* and endosymbionts imparted adaptive benefits to their interactions, including improvements in transport systems, antibiotic resistance, stress responses, cell signaling, and bacterial virulence (68, 69). This study found that ARG transfer between *Acanthamoeba* and *Burkholderia* may have enriched the *Acanthamoeba* genome with antibiotic resistance, particularly *amrA* and *amrB*, making *Acanthamoeba* potentially less susceptible to neomycin and azithromycin. This may be attributable to the close and long-term symbiosis of *Burkholderia* with *Acanthamoeba*, as well as sustained drug exposure. And the transfer of ARGs from *Burkholderia* to *Acanthamoeba* involved unequal functional tendencies, which may be related to an environment that facilitated the adaptation of *Acanthamoeba* to ocular broad-spectrum antibiotic therapy. From the *Acanthamoeba* perspective, this analysis provided additional evidence that ARGs originating from *Burkholderia* may contribute to the genetic diversity of *Acanthamoeba* resistance genes, potentially informing clinical dosing strategies. Although this analysis is based on studies between *Burkholderia* and *Acanthamoeba*, it provides a practical reference for us to explore the mechanism of action of AK, and more endosymbiont analyses such as *Pseudomonas* will be carried out in the future to refine our findings. Patient prognosis with AK generally hinged on multiple factors, including the pathogenicity of the causative strain and the timeliness of diagnosis and treatment. However, this finding suggested that intracellular endosymbiont species of *Acanthamoeba* also influenced disease progression, a factor that clinicians should consider. Furthermore, the study revealed that the extent and characteristics of gene exchange were confined to the same family, with notable differences between isolates and species families, indicating strain variability and species specificity in gene exchange between *Acanthamoeba* and endosymbionts. This aligned with the findings of prior research (28). Most candidate genes for HGT appeared to have undergone significant internalization, with *Acanthamoeba* incorporating them into its established transcriptional programs (68). Of course, future research involving more data on clinical outcomes, drug sensitivity, and experimental analysis will be needed to validate these findings.

### Conclusions

In this study, we provided a comprehensive characterization of the functionally annotated differences between different sources of *Acanthamoeba*. In addition, this work offered a better understanding of the species diversity and functional potential of endosymbionts. It highlighted that the existence of potential horizontal ARG transfer events between ocular *Acanthamoeba* and endosymbionts may lead to specific drug resistance of *Acanthamoeba* associated with endosymbiont. This sympatric lifestyle fostered an environment conducive to the exchange of genetic material between different organisms, thereby enriching the gene pool and contributing to the persistence and evolution of drug-resistant traits.

## MATERIALS AND METHODS

### Strains

In this study, a total of 48 *Acanthamoeba* whole genomes were analyzed; 19 of these were clinical pathogenic *Acanthamoeba* isolates from corneal AK ulcer patients, provided by the Department of Laboratory Research at Eye Hospital of Shandong First Medical University, Shandong, China. Additionally, we downloaded publicly available genome sequences of 29 *Acanthamoeba* strains from the GenBank database of the National Center for Biotechnology Information (NCBI) (https://www.ncbi.nlm.nih.gov/datasets/genome/). Table S3 provides detailed information. These 48 strains were isolated from various countries, including China, the USA, Germany, India, and other countries.

Twenty-nine *Acanthamoeba* strains were selected to represent known sources, of which 7 were isolated from the eye, 10 strains were isolated from the environment, including water, soil, and coprolites, and 12 were isolated from other sources, including model strains, brain, BeWo human choriocarcinoma cells, primary monkey kidney tissue culture, and uncertain origin.

### Culture, DNA isolation, and 18S rRNA gene sequence typing

Nineteen clinical *Acanthamoeba* isolates were inoculated onto non-nutrient agar plates composed of Page’s modified Neff’s amoeba saline (PAS: 1.2 g of NaCl, 0.04 g of MgSO_4_ × 7H_2_O, 0.03 g of CaCl_2_, 1.42 g of NaHPO_4_, and 1.36 g of KH_2_PO_4_ in 1 L ddH_2_O) and agar (70). This was supplemented with *Escherichia coli* ATCC25922 as a food source for the *Acanthamoeba* isolates. The cultures were incubated in a wet box at 30°C for constant temperature proliferation. Plates were monitored daily under a microscope to observe isolates’ growth, and trophozoites were harvested during the logarithmic growth period. Genomic DNA of 19 clinical *Acanthamoeba* isolates was extracted from the obtained trophozoites using a DNA extraction kit (Qiagen) (71, 72).

The 18S rRNA gene sequences were amplified using polymerase chain reaction with primers JDP1 and JDP2 (JDP1: 5′-GGCCCAGATCGTTTACCGTGAA-3′; JDP2: 5′-TCTCACAAGCTGCTAGGGAGTCA-3′) followed by DNA sequencing (72). These sequences underwent homology comparison based on the nt database using BLASTn software to identify the isolates’ genus. Phylogenetic reconstruction involved genotype or subtype sequences of *Acanthamoeba* T1–T23, alongside the 18S rRNA gene sequences obtained in this study for genotyping. Alignment of nucleotide sequences was executed using the MAFFT program (73), and a phylogenetic tree was constructed using FastTree with the maximum likelihood algorithm (74). Visualization and beautification of the phylogenetic tree were done by iToL v6 (75).

### Whole-genome sequencing, genome assembly, gene predictions, and functional annotations

The genomic DNA of the 19 *Acanthamoeba* isolates was sequenced on an Illumina Novaseq 6000 platform by Novogene Co., Ltd., Beijing, China, generating 150 bp paired-end reads. The high-throughput sequencing strategy was Illumina PE150, and the sequencing principle was Sequencing by Synthesis. Initially, the quality of raw data was assessed using FastQC (v0.11.9), and the raw data were trimmed using Trimmomatic-0.39 (76) by removing adapter sequences and filtering low-quality reads. In addition, high-quality sequence reads from each isolate were *de novo* assembled using SPAdes (v3.15.5) (77), while QUAST (v4.6.0) (78) was employed to evaluate the quality of each genome assembly.

Gene prediction was conducted on all genomes, including the 19 ocular-sourced *Acanthamoeba* assembly genomes obtained by sequencing in this study and 29 *Acanthamoeba* genomes downloaded from the NCBI Genbank database, by AUGUSTUS (v3.4.0), a software-optimized for eukaryotic genomes. To annotate functionally, Eggnog-mapper (v2.1.0) of the Diamond parameter (79) was utilized to assign functions to *Acanthamoeba*-predicted proteins via sequence-similarity search with orthologous genes in public databases, including COGs database and KEGG database.

### Pan-genome analysis

Pan-genome analysis of genomes of *Acanthamoeba* from different sources was carried out by BPGA v1.3 (80), with the default parameters and 50% sequence identity as the cutoff for clustering identity was applied to USEARCH.

Further analyses, including gene accumulation curves and the composition of core, accessory, and unique sequences, were based on the initial findings. After the pan-genomic analysis, the COG categories and KEGG pathways of core, accessory, and unique sequences were further validated through functional annotation using Eggnog-mapper (v2.1.0) in the COG and KEGG databases.

### Analysis of antimicrobial resistance genes

Scaffolds that may have originated from *Escherichia coli* ATCC 25922 were removed. ARGs were identified using Resistance Gene Identifier v6.0.2 against CARD (35).

### Taxonomical distribution of endosymbionts and horizontal transfer of antimicrobial resistance genes

The taxonomical distribution of endosymbionts was identified based on the best hits between *Acanthamoeba*-predicted protein sequences and proteins from endosymbionts, utilizing a BLASTp search of the Diamond parameter against the nr database (81). Networks between protein sequences from endosymbionts and ARGs from the assembled genome sequences of *Acanthamoeba* isolates were generated by Circos. *Acanthamoeba* ARGs that had the best hits with *Burkholderia* protein sequences were used as queries to search into the nr database by BLASTp. Alignments of homologous amino acid sequences were performed using the MUSCLE program. Phylogenetic trees, based on the *Acanthamoeba* ARGs sequences and the endosymbionts homologs with the highest hit rates, were constructed using FastTree with the maximum likelihood algorithm. Subsequently, each phylogenetic tree for horizontal ARG transfer candidates was manually checked, and only those showing a clear pattern of horizontal ARG transfer were accepted. Finally, *Acanthamoeba* ARGs sequences and the *Burkholderia* homologs with the highest hits underwent analysis in a mosaic format. This result was derived using a BLASTp search based on the nr database, revealing the best match with *Burkholderia* homologs within a 40 amino acid window. Mosaic visualization was performed using Circos.

### Statistical analyses

The significance of abundance in core, accessory, and unique genes within COG categories was analyzed using Fisher’s exact tests and involved the FDR correction of *P* values. All statistical analyses were performed using the R package (v4.3.2). A *P* value <0.05 was considered statistically significant.

## Data Availability

The whole-genome sequencing data have been submitted to the NCBI Sequence Read Archive (SRA) under BioProject accession number PRJNA1125420.

## SUPPLEMENTAL MATERIAL

Table S1 msphere.00548-24-s0001.xlsx The genomic characteristics of 19 pathogenic AK strains.

Table S2 msphere.00548-24-s0002.xlsx List of *Acanthamoeba* ARGs shared with endosymbionts.

Table S3 msphere.00548-24-s0003.xlsx Source information of 29 downloaded *Acanthamoeba* whole genome sequences.

Table S4 msphere.00548-24-s0004.xlsx KEGG functional annotation.

ASM does not own the copyrights to Supplemental Material that may be linked to, or accessed through, an article. The authors have granted ASM a non-exclusive, world-wide license to publish the Supplemental Material files. Please contact the corresponding author directly for reuse.
